# Quotas and Gender Competence: Independent or Complementary Approaches to Gender Equality?

**DOI:** 10.3389/fsoc.2021.740462

**Published:** 2021-08-27

**Authors:** Angela Wroblewski

**Affiliations:** Institute for Advanced Studies, Research Group Higher Education Research, Vienna, Austria

**Keywords:** gender equality policy, quota, higher education institutions, gender competence, Austria

## Abstract

Austrian gender equality policy in higher education is characterized by the successful implementation of a comprehensive set of gender equality policies and persistent gender imbalances. After the introduction of a legal quota for university bodies, for instance, female representation in decision-making bodies increased significantly within a short period of time. However, this did not lead to a cultural change or the abolishment of barriers to women’s careers. Research has attributed this paradoxical situation to a lack of reflexivity because the current gender equality policies do not force institutions or individuals to challenge traditional practices, which are perceived to be merit-based and therefore gender neutral. To overcome this paradox, the Austrian Federal Ministry of Education, Science, and Research launched a policy process aimed at strengthening gender competence in all higher education processes—management, administration, teaching, and research. This paper provides a critical discussion of the Austrian quota regulation and its implementation. It also introduces the concept of gender competence and outlines the underlying assumptions as to why the new policy is expected to contribute to change. Following a critical reflection on these assumptions, the paper also discusses how existing steering instruments have to be adapted to support individual and institutional reflexivity.

## Introduction

The Austrian university sector is dominated by public universities and the ideal of open access to university education (universities should be open for all talented students). Hence, the major source of funding is the Austrian state; there are no or only very low fees for students. Although universities are publicly funded, they enjoy far-reaching autonomy in terms of budget distribution, staffing, strategic planning, and governance (Höllinger and Titscher, 2004). The relationship between the state and the universities is based on performance agreements, which define the budget of a university as well as its main duties for a 3-year period (Biedermann and Strehl, 2002). Universities report their performance to the Federal Ministry of Education, Science and Research on an annual basis in the form of an intellectual capital report, which is based on a set of key indicators (e.g., student and staff numbers, courses offered, third party funding).

The character of the higher education system in Austria is shaped by the Humboldtian tradition. Academic careers are thus structured in the typical pattern for the Humboldtian university, which is based on unity in teaching and research, freedom of study, and corporate autonomy for universities despite their being funded by the state (Münch, 2007). This model is characterized by a strict hierarchical division between full professors and academics at lower stages of their careers. A successful academic career leads to a professorship, and academics remain dependent on the chair to which their position is assigned until this is achieved (Pechar and Andres, 2015). This career model is highly compatible with the ideal notion of a good scientist developed by Max Weber in the early 20th century (Gerth and Wright Mills, 1946), whereby an ideal scientist is able to devote his life entirely to science without restrictions due to other commitments like care responsibilities. This perception was developed at a time when women were formally excluded from universities and refers to a typical male career. Criteria to identify excellence are still derived from it, and it also defines selection practices and procedures in academia.

The combination of the Humboldtian university tradition and the broad patterns of female labor market participation significantly limits the prospects for equal career outcomes for women in academia. In general, women’s labor market participation in Austria still relies on the conservative welfare state model, which is characterized by a modified male breadwinner (Crompton, 1999; Buber-Ennser, 2015; Behrens et al., 2018). This supports women working part-time to reconcile their unpaid and paid work–in the labor market in general as well as in academic professions and despite the fact that highly qualified women tend to return earlier after maternity leave, postpone their family planning to suit the dynamics of academic careers, and on average have fewer children (Beaujouan and Berghammer, 2019). Working part-time also limits women’s career prospects in academia. This is mainly due to the assumption that high-profile jobs or management positions cannot be accomplished on a part-time basis. Consequently, more women than men work part-time in higher education and research. According to the recent She Figures (European Commission, 2019a), 22% of women and 11% of men work part-time in higher education and research in Austria.

Beginning with the education expansion in the 1960s, increasing numbers of women gained the necessary qualifications to enroll at university. The development of female participation in higher education in Austria is no exception: since the turn of the 21st century, more women than men have enrolled at university, and women now also make up the majority of graduates. However, the “leaky pipeline” phenomenon (Blickenstaff, 2005; Connolly and Fuchs, 2009; Emerek and Larsen, 2011) is very persistent, with the share of women decreasing in higher status positions, and the share of female professors remaining below the European average (23% in 2016, European Commission, 2019b). Gender-segregated degree choice is another very persistent phenomenon (European Commission, 2019a): in Austria, women are overrepresented in the education sector (share of women among PhD graduates in 2016: 76%) and underrepresented in the engineering, manufacturing, and construction sectors (share of women among PhD graduates in 2016: 26%).

In the early 2000s, the organization of universities in Austria was fundamentally reformed. The new organizational law, the Austrian Universities Act 2002, gave universities autonomy over budgetary and personnel matters (Höllinger and Titscher, 2004). The Act also constitutes the legal foundation for gender equality in higher education and formulates gender equality and equal opportunities as guiding principles (§2) and duties (§3) of universities. Each university has to establish an equal opportunities working group which is responsible for preventing discrimination in appointment procedures (§42), set up an organizational unit responsible for the co-ordination of activities relating to equal opportunities, the advancement of women and gender research (§19) and publish a female advancement plan and a gender equality plan (§20) as part of the university statute. The 2009 Amendment to the Universities Act also establishes a quota regulation for the composition of university bodies (Hölzl and Neuwirth, 2020).

Austria has a long tradition of gender equality policies in higher education that started in the 1980s (Schaller-Steidl and Neuwirth, 2003). The initial policy mix comprised measures to support qualified women in higher education (first among students and later among professors), prevent discrimination and institutionalize women’s and gender studies, and was developed prior to the universities gaining autonomy. It was subsequently expanded in the late 1990s when Austria began implementing gender mainstreaming in higher education (Wroblewski et al., 2007). The policy mix was based on Rosabeth Kanter’s (1977) theory of the critical mass and the assumption that an increasing participation of women in higher education would lead both to an increasing share of women in top positions as well as to cultural change.

In the last decades, university organizational reforms have seen gender equality goals introduced into steering instruments (Ulrich, 2006) and the related monitoring instruments in higher education (Eckstein, 2017; Wroblewski, 2017). Each university in Austria formulates its own gender equality goals and measures in its performance agreements. Since universities in Austria gained their autonomy, a heterogeneous bundle of gender equality measures has emerged, albeit with different priorities, target groups, and intensities (Wroblewski and Striedinger, 2018). To monitor progress towards gender equality goals, gender monitoring was introduced based on the obligatory annual intellectual capital reports submitted by the universities. This gender monitoring contains indicators on the representation of women and men in all areas and at all hierarchical levels (including management and decision-making bodies and committees), the career advancement opportunities open to women and the gender pay gap.

Universities are not the only establishments in Austria that are required to formulate concrete gender equality objectives. Since the introduction of the outcome-oriented approach for government spending in Austria, each Federal Ministry is obliged to formulate corresponding targets (including one gender equality objective^1^). In this context, the Federal Ministry of Education, Science and Research developed corresponding gender equality goals that are incorporated into the university performance agreements. Austria is also committed to the current gender equality policy in the European Research Area (ERA) and has included the objective to achieve gender balance in decision making in its ERA Roadmap 2016–2020 (Federal Ministry of Science, 2016).

The paper describes the Austrian quota regulation and women’s representation in decision making positions as well as a recent policy aiming at strengthening gender competence in higher education processes. These policies and achieved or expected results regarding gender equality are discussed from a feminist institutionalist perspective (Kenny, 2014; Krook and Mackay, 2011; Mackay et al., 2010) and a practice theoretical point of view (Schatzki, 1996; Schatzki, 2003). Hence, the effective implementation of gender equality policies to achieve cultural change requires a change of organizational gendered practices (Acker, 1990; Martin, 2003; Martin, 2006). For example, regulations aiming at a reduction of implicit gender bias in procedures only contribute to change if they are known, accepted and followed by relevant stakeholders.

## Quotas for University Bodies

Although women conquered universities in Austria at student and researcher level, they initially remained excluded from top positions like full professorships and top management (Wroblewski and Striedinger, 2018). Hence, after the turn of the century it became clear that the assumption on which gender equality policies have been based since the 1990s does not hold. The development of first gender equality policies in Austria followed Rosabeth Kanter’s hypotheses that after a critical mass of women entered the system, culture will change, and women will find their way into top positions (Schaller-Steidl and Neuwirth, 2003). To rectify this situation and to increase women’s representation in decision making, a quota for university bodies (rectorate, university council and senate) was introduced in 2009 through an amendment to the Universities Act 2002 (Schulev-Steindl, 2010).

Along with the council and the senate, the rectorate is the highest management body in a university. The rectorate manages the university and represents it in the outside world. The rector is the head of the rectorate and also acts as its spokesperson (§ 22, Universities Act 2002). Rector positions must be publicly advertised. A rector is appointed by the university council for a period of 4 years from a shortlist of three candidates proposed by the senate. Vice rectors are appointed by the university council on the recommendation of the rector following a senate hearing. Their term of office corresponds to that of the rector.

The function of the university council is defined in the Universities Act 2002 (§21) and corresponds roughly to that of a corporate supervisory board. A university council consists of either five, seven, or nine members (the actual size is determined in each case by the university’s founding convention). Two, three, or four of the members (depending on the size of the council) are elected by the senate, and the same number are appointed by the Federal Government on the proposal of the Federal Minister of Education, Science and Research. The remaining member is appointed by mutual agreement by the members of the university council.

The university senate is made up of representatives of professors, scientific non-professorial staff, general university staff, and students. The senate is dominated by professors, who represent 50% of its members. Students make up 25% of senate members. The tasks of the senate include the approval of the university’s development and organizational plans, the preparation of proposals for the election of the rector (together with the university council), the acceptance of curricula and the adoption of the university’s statutes.

### The Austrian Quota Regulation

Austrian equality law establishes a general duty on the part of the public sector to give preference to female candidates as long as the share of women in the respective category has not reached 50% (Federal Act on Equal Treatment in the Public Service, Bundesgleichbehandlungsgesetz § 11). In line with this regulation, the quota regulation for decision-making bodies at Austrian universities was introduced in 2009 through an Amendment to the Universities Act 2002. University bodies like the rectorate, council, senate, and all commissions installed by the senate are required to fulfill a quota of female members (Schulev-Steindl, 2010). Until 2014, the quota regulation foresaw that all university bodies had to consist of at least 40% women. In 2014, the quota was increased to 50%.

Since the law also contains sanctions for non-compliance, the quota regulation can be interpreted as a strong one (Guldvik, 2011). If a university body does not fulfill the required quota, the equal opportunities working group may request a new composition of the body, which makes all decisions taken by it invalid. The equal opportunities working group may also explicitly agree to a university body not fulfilling the quota based on a justification report provided by the authority responsible for its setup (e.g., if there are no women professors available or willing to join it). At some universities, the working groups for equal opportunities have also stated that they will not object to imbalanced committees if their members can demonstrate competencies in regard to gender issues (Wroblewski, 2015).

The quota regulation aims at increasing women’s participation in decision making and not at gender balance. This is evident in the formulation used in the law, which stipulates a quota of at least 50% women in university bodies. According to the legal formulation, there is no problem with an overrepresentation of women. The law does not talk about abolishing a gender bias in decision making related to gendered decision-making criteria. Nevertheless, in the parliamentary debate on the quota regulation it was assumed by representatives of most political parties that an increasing participation of women in decision making would lead to more gender-fair decisions (Wroblewski, 2019a). Referring to the work of Sarah (Childs and Krook, 2008), the Austrian quota regulation focuses explicitly on numeric representation, i.e., the number of female representatives, and aims only implicitly for a stronger attention to women’s concerns or a reduction of a gender bias in decision making processes and criteria (substantive representation).

### Women’s Representation in Decision Making

**FIGURE 1:**
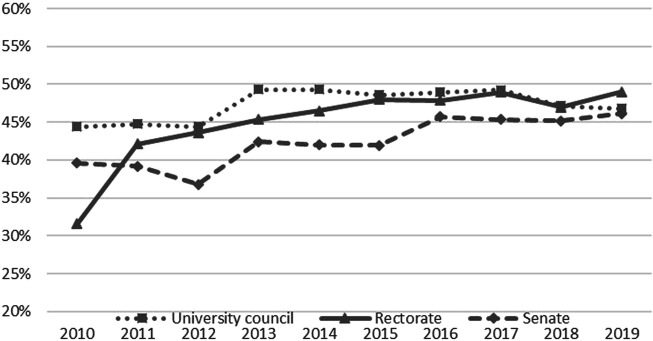
Share of women in rectorates, senates, and university councils 2010-2019.

The implementation of the quota regulation is monitored by the Federal Ministry of Education, Science and Research. Data on the composition of university bodies is available for the period since 2010. Women’s representation increased significantly immediately following the introduction of the quota regulation (see Figure 1). The share of women in rectorate positions increased from 22% in 2005 to 49% in 2019. The most significant increase was seen in 2011, when the share of women in rectorates increased by almost 10 percentage points (from 32% in 2010 to 41% in 2011). In other words, only 2 years after the introduction of the quota regulation, the overall share of female rectorate members lay at over 40%. The development in women’s participation in university councils started from a higher level and already reached parity in 2013. In 2018 and 2019, the share of women among council members decreased to 47%. Compared to rectorates and university councils, senates appear to face more difficulties in meeting the quota. This is due to a combination of the underrepresentation of women among full professors and the dominance of professorial members in the senate. The share of women among senate members varies between 37 and 46%.

The figures presented above show the average over all universities. Since 2013, almost all university councils and rectorates have fulfilled the quota regulation. As already mentioned, the situation is different when it comes to senates: since 2013, about half of the senates have met the quota.

A more differentiated look at the composition of university bodies reveals that women’s representation is higher among ordinary members than in leading positions (see Table 1). In 2010, although women already represented about half of ordinary council members, only 23% of university councils were headed by a woman. In 2019, one third of university councils were headed by women, and gender parity had been reached among members.

**TABLE 1:** Share of women in university councils, rectorates, senates, and senate committees (2010–2019).

	2019 (%)	2018 (%)	2017 (%)	2016 (%)	2015 (%)	2014 (%)	2013 (%)	2012 (%)	2011 (%)	2010 (%)
University council total	47	47	49	49	49	49	49	44	45	44
Head	36	32	41	45	45	45	45	27	23	23
Other members	49	50	51	50	49	50	50	48	49	48
Rectorate total	49	47	49	48	48	46	45	44	42	32
Rector	29	33	33	38	36	27	24	20	19	5
Vice rectors	55	50	53	51	51	52	51	50	49	40
Senate total	46	45	45	46	42	42	42	37	39	40
Head	36	18	18	18	14	18	18	27	27	27
Other members	47	46	47	47	43	43	44	37	40	40
Habilitation committee	43	42	42	44	42	40	38	39	37	35
Appointment committee	44	45	44	43	42	41	42	43	40	34
Curricular committee	46	43	43	44	40	40	40	38	37	38
Other senate committees	52	51	53	52	46	46	46	44	44	47

Source: Repository of the federal ministry of education, science and research, www.unidata.gv.at.

The development in rectorates is fairly similar: as far as vice rectors are concerned, women already accounted for 40% in 2010 and have made up the majority since 2012. While women are still underrepresented among rectors, the number of female rectors has at least risen from its initial low level. In 2007, there was just one female rector in Austria; from 2011 onwards, more and more women were appointed to this role, with their share reaching its peak in 2016 at 38% (2019: 29%).

Compared to university councils and rectorates, senates show a pronounced difference between heads and members. The share of women among senate heads varies between 14% (2015) and 36% (2019) with upward and downward trends. The share of female senate members, in turn, varies between 37% (2012) and 47% (2019).

We can therefore conclude that Austria’s introduction of a quota for decision-making bodies in universities has had the desired result. The quota forces those who are responsible for the composition of a body to search for qualified women members. And as the results show, they have been successful in doing so. However, some barriers do still exist as women remain underrepresented among heads of university bodies. Interestingly, a recent empirical study on women in university management shows that, on average, women take up a position as rector or vice rector at a younger age than their male counterparts and are less likely to have held a full professorship prior to entering the rectorate (Wroblewski, 2015). Hence, their situation differs: men often hold rectorate positions in the final stages of their academic careers and retire after their term in office. Women, in contrast, hold this position earlier in their careers but do not have the option to return to a chair afterwards.

In contrast, it should however be noted that the increase in the share of women among full professors in Austria has been far more moderate (from 16% in 2006 to 25% in 2018). When compared with other countries, Austria ranks above the EU average for female heads of universities yet below the EU average for the share of women in Grade A positions (European Commission, 2019b). The moderate increase in the share of women among full professors point out the limits of the quota regulation for decision making bodies and illustrates the discrepancy between numeric and substantive representation. It shows that an increasing share of women in decision making does not automatically lead to an adaptation of biased decision making criteria or processes.

### Assessment of the Quota Regulation and its Implementation

In the public debate, increasing female participation in rectorates is seen as progress towards gender equality. While this assessment is strengthened by the fact that women are not only assigned “soft” rectorates (e.g., responsibility for student affairs or human resources), they are nonetheless still underrepresented in vice rectorates responsible for research, most of which are headed by full professors.

In the literature, increasing participation of women in gatekeeper positions (Husu, 2004) is also identified as a potential for cultural change, since it is often assumed that women in decision-making positions will promote women and put women’s issues on the agenda (EC 2004). But does this prove true in practice? To what extent does the increasing participation of women in decision-making bodies contribute to cultural change? Dalhoff (2021) recently discussed the limited effects of gender equality polices in the past decades due to a lack of reflexivity not only regarding causes of inequalities but also regarding gender equality objectives–including the objective of cultural change. She calls for cultural change in terms of a change in disciplinary cultures and in university processes and structures. A recent study among Austrian female rectors and vice rectors sought to answer the question to which extend women in rectorate positions feel responsible for gender equality in general and cultural change specifically (Wroblewski, 2015).

In some cases, women head the vice rectorate that is, formally responsible for gender equality, diversity or the advancement of women at their university. All of these women embrace this responsibility and see these topics as priorities for the rectorate. They also interpret the reference to gender equality, diversity, or advancement of women in the name of their vice rectorate as a demonstration of the rectorate’s commitment to these topics. However, while most of them did not actively seek this responsibility, they recognize and accept its importance.

Those female vice rectors who are formally assigned this competence pursue different priorities in this regard during their terms of office (e.g., advancement of women, involvement of fathers in unpaid work). These priorities and the concrete measures taken depend both on the level of importance accorded to gender equality at their university when they were appointed to the rectorate as well as on their own corresponding experience. Those of them who work at universities with longer traditions of gender mainstreaming and the advancement of women and/or those with expertise in these fields (e.g., through participation in a working group for equal opportunities or knowledge of gender research) build on the structures that are already in place and work closely with the corresponding experts in their organizations.

At the other end of the scale are the female vice rectors who are not–and did not want to be–formally responsible for gender equality, advancement of women or diversity. These women also formulated clear reservations towards positive action or specific measures (e.g., the quota regulation) and assigned the responsibility for gender equality to experts in the organization. Consequently, they did not consider gender equality to be a main task or priority of the rectorate and considered other topics to be more relevant than gender equality.

Formal competence or non-competence for the advancement of women, gender equality, and/or diversity also cannot be linked directly to a feminist background or gender expertise (or lack thereof). While most of the participants in the study who are formally responsible for these topics do have a feminist background, some of those who are not are also feminists and/or gender experts (Wroblewski, 2015, 8). Regardless of their formal competence, those who see themselves as feminists all seek to change the structures and processes in their area of responsibility and take a closer look at the actual situation for both women and men. They also realize that people expect female managers to adopt a different style of management to men.

They do, however, also take issue with the general assumptions that female rectors or vice rectors are frequently confronted with. These include, for instance, the assumption that the gender equality problem is “resolved” with the appointment of a woman or the expectation that women in rectorate positions will change the system and “do something for women” (like putting women’s interests on the agenda and promoting qualified women).

To conclude, experiences with the Austrian quota regulation show that increasing female participation in decision making does not automatically initiate cultural change. Moreover, male members of the rectorate ascribe gender competence and the responsibility for gender equality to women. Women with a feminist background who hold a vice rectorate position which focuses on gender equality and/or diversity formulate a gender equality goal for their term in office and aim at initiating sustainable change. They do so by adapting decision-making processes or criteria, putting women’s issues on the agenda or actively promoting women. However, since gender expertise is not yet included as a selection criterion for rectorate positions, it does not seem realistic to rely purely on feminists in rectorate positions to initiate cultural change.

## Gender Competence Policy

### Description of the Policy Process

After the introduction of the Austrian quota regulation, women’s participation in decision making increased significantly. However, this did not initiate cultural change for several reasons. First, and probably most importantly, cultural change has not been explicitly formulated as a goal in the quota regulation context. Second, selection criteria for members of university boards do not include gender competence or experience with gender equality policies if gender equality or diversity is not the focus of the actual vice rectorate. Third, it is generally expected that women in decision-making positions will take responsibility for gender equality. Consequently, most men either do not feel responsible for gender equality or don’t see any need for action in their field of responsibility.

To complement the existing gender equality policy mix and to increase their impact, the Austrian Federal Ministry of Education, Science and Research initiated a political discourse on gender competence in higher education. The Federal Ministry of Education, Science and Research assumes that building up gender competence will also strengthen the implementation of existing policies and thus contribute to cultural change in higher education institutions. “Those responsible for the cultural change are members of higher education institutions, whose actions shape the structures and processes in a gender competent way. It is therefore indispensable that the higher education institutions take a clear stance on the necessary change of culture and implement the recommendations to strengthen gender competence.” (Federal Ministry of Education, Science and Research, 2018, 7). The process started in October 2016 with the establishment of a working group^2^ set up by the Austrian Higher Education Conference^3^. This working group was moderated by a departmental head at the ministry and was given the mandate to develop recommendations to raise gender competence and awareness for gender diversity among managers of higher education institutions. These recommendations should be concrete, action-oriented and address all relevant stakeholders (individuals and committees). Targets and background information should be provided for each specific recommendation.

As a first step, the working group developed a definition of gender competence that distinguishes gender competence from gender expertise and follows both the gender mainstreaming tradition (Rees, 1998; Holzleithner, 2004) and a pedagogical concept of competence (Klenk and Langendorf, 2016).

“Gender competence comprises of the fundamental recognition of the relevance of gender attributions in one’s own work and sphere of influence (knowledge). This recognition is connected with the willingness (desire) and ability to deal with these issues in day-to-day work and study life–if necessary, supported by gender experts and with knowledge from gender theories–and to take action based on this knowledge (skills). Recognition, discussion and action are subject to a constant process of reflection (reflection).” (Federal Ministry of Education, Science and Research, 2018, 33).

Gender competence also requires the ability to act on the basis of this reflection and set actions which tackle these gender attributes and their gendered consequences. Hence, gender competence requires constant reflection on the gender dimension in one’s own field of work. Gender competence is a basic competence that all stakeholders should have. University lecturers, researchers, administrative staff, managers, and students should all be gender competent. Gender expertise, in contrast, is defined as profound knowledge of gender theories and/or experience with gender mainstreaming implementation processes.

The working group prepared a position paper containing a total of 36 recommendations for building up gender competence and ensuring its consideration in all higher education processes and tasks. These recommendations are divided into four sections–gender-competent management, administration, teaching, and research. Each of these sections explains the central idea for this particular area and includes 2 to 18 recommendations–along with details of the rationale behind them (i.e., why they are relevant for gender equality), the responsible stakeholders and the groups who will benefit.

The guiding principle for the “gender competent management” section in the position paper assigns university management the duty to “make use of and develop opportunities for change and innovation, and for quality assurance.” (Federal Ministry of Education, Science and Research, 2018, 9). The working group formulated 18 recommendations for this section. Among others, they recommend formulating an explicit commitment to strengthening gender competence, setting concrete objectives, and implementing measures. They also recommend integrating this commitment into existing strategic documents and assigning responsibility for strengthening gender competence to one member of top management (vice rectorate).

Recommendation six focuses on gender competence in decision-making bodies. “The working group recommends that higher education institutions include gender competence into the requirement profile for university commissions/committees.” (Federal Ministry of Education, Science and Research, 2018, 12) The university management is responsible for implementing the recommendation, while committee members and (future) applicants will benefit from the outcome. The working group justifies this recommendation by noting that committees and bodies in higher education institutions take numerous personnel and strategic decisions. Hence, they are of central importance when it comes to avoiding gender-biased decisions. Higher education institutions could offer training measures for entire committees or individual committee members to teach them about gender competence and its relevance for appointment procedures. In order to act in a gender competent manner, the whole committee–and not just individual members–has to be gender competent. The recommendation closes by referring to concrete training measures that have already been implemented at some universities in Austria (e.g., anti-bias training) as well as to existing guidelines for gender-fair appointment procedures.

### First Implementation Steps

The members of the working group used the slogan “Because it is 2019!”^4^ as a springboard for their discussions and recommendations. This slogan expresses their commitment to supporting gender equality in higher education institutions. However, the policy paper, which was adopted by the Austrian Higher Education Conference in early 2019, is first and foremost a declaration of political will. To achieve change it is necessary to embed it in a policy discourse and to develop accompanying measures which support the implementation of recommendations. The policy paper has been presented and published, which is a precondition and the starting point for a policy discourse.

The Federal Ministry of Education, Science and Research committed itself to supporting a policy discourse on gender competence in higher education processes. As a first step in this direction, the ministry conducted a survey among Austrian higher education institutions to determine which of the recommendations had already been implemented in the past, which concrete measures are in place and where institutions themselves see a need for action (Federal Ministry of Education, Science and Research, 2021). Almost three out of four higher education institutions participated in the survey (return rate: 73%). All the universities who participated in the survey already follow at least one of the 28 recommendations that address universities. The numbers of recommendations already implemented vary between 1 and 27. On average, universities have already implemented measures relating to 16 of the recommendations. However, the survey results only indicate the availability of concrete measures which address the recommendations; they do not show whether these have actually been implemented. The respondents were also asked to name the hindering factors they face in the context of strengthening gender competence. The most important such hindering factors are a lack of expertise, wrong self-assessment and lack of dedicated resources.

The survey results also indicate that most universities informed relevant stakeholders about the recommendations of the position paper by sending it to them by e-mail. 44% of universities organized internal events to present and discuss the recommendations. Hence, the majority of universities did not assume an active role in discussing the recommendations internally. Given this inactivity, it is extremely important that the Federal Ministry of Education Science and Research had committed itself to supporting a policy discourse. Based on the survey results, the ministry organized a 1-day networking meeting on October 14, 2020. The meeting took place online due to COVID-19 restrictions, was attended by more than 100 people and included a total of eight workshops with experts from Austria and Germany that focused on good practice examples and topics that had been identified as relevant. The workshops addressed different target groups relevant for the successful implementation of gender equality policies (members of rectorates, gender equality officers, members of curricula commissions, quality assurance officers, etc.).

### Assessment of the Policy Process

Given the logic of existing steering instruments in higher education policy, concrete objectives now need to be formulated and used as the basis for the development, implementation, monitoring, and evaluation of measures. The ministry is asking universities to include measures aimed at strengthening gender competence in higher education processes in their performance agreements. Accordingly, the topic is addressed in the negotiations that accompany these performance agreements, and universities will include such measures in their performance agreements. However, this does not guarantee that the measures will be implemented effectively and contribute to real change. There is still a risk that measures remain paper tigers and do not gain relevance in everyday practices. The ministry has therefore committed itself to continue organizing networking events to complement these activities and support a political discourse on gender competence. These networking activities should lead to a common understanding of the relevance of gender competence and should focus on exchange of experiences and mutual learning, e.g., regarding good practice measures. They could also establish the basis for joint or common initiatives.

A crucial aspect of the plans outlined above is how seriously the goal of strengthening gender competence in higher education processes will have to be incorporated into existing steering instruments. If the process only requires simply mentioning measures, the instrument will remain ineffectual. If the formulation of concrete, ambitious, realistic, and measurable goals at an institutional level is required, related monitoring indicators to measure gender competence in higher education institutions will have to be developed. To date, the monitoring system for the performance agreements does not contain any indicators that focus on gender competence. Given the complexity of the gender competence construct, the development of such indicators will be a challenging endeavor. But it will also constitute an essential step towards cultural change and provide important input for the discourse on gender competence in academia.

## Discussion and Recommendations

With the introduction of a statutory quota regulation, Austria succeeded in significantly raising the participation of women in university management functions in a short period of time. However, the positive trend in women’s numeric representation in decision making did not initiate cultural change. This conclusion is supported by the stable representation of women among full professors. Thus, gendered appointment procedures and selection criteria (Van den Brink, 2009; Van den Brink et al., 2010) have not been altered.

When the quota regulation was debated in parliament, it became evident that its primary aim lay on increasing women’s representation in decision making in numeric terms. It was assumed that doing so would lead to more women-friendly or gender-fair decisions (Wroblewski 2019a). Thus, it was assumed that numeric representation automatically leads to substantive representation or cultural change. This tacit expectation harbors the risk that women in rectorate positions will be automatically assigned responsibility for gender equality and thus also saddled with the corresponding load. Helen Peterson (2015) describes this risk of overload as a potential exploitation of women “in the name of gender.” Cultural change, in contrast, was never formulated as an explicit goal.

While this positive development in women’s representation in decision making was the result of the active search for qualified women to fill the positions, gender expertise, or competence in gender equality appear to have played only a limited role in their selection. As a consequence, women who distance themselves from gender equality objectives or deny the need for cultural change also found their way into top management positions. Hence, the increasing level of female participation in top positions indicates first and foremost that access barriers for women to these positions have been successfully dismantled.

Given the above, it is not surprising that the quota has had only limited effect on cultural change. As long as women did not actively pursue the objective of structural change–in most cases due to their feminist background–it was possible to continue with a proforma implementation of gender equality policies. Austrian higher education policy addressed this problem with its gender competence policy, which aims at strengthening the effectiveness of existing gender equality policies and can be interpreted as a renewal of the gender mainstreaming strategy (Rees, 1998). All actors should consider gender issues in their own sphere of responsibility and their everyday work processes.

To exploit the potential of the gender competence strategy for cultural change, it is recommended 1) that an explicit cultural change objective is formulated at institutional and political level and 2) that this objective is integrated into existing steering instruments. Both approaches are challenging and require a further development of existing gender equality policies.

As already described above, most universities in Austria have formulated cultural change as part of their gender equality strategy. However, their commitment to cultural change often remains solely at a rhetoric level and is not linked to concrete objectives. This missing concretization of the cultural change objective is difficult in the context of steering instruments which are based on quantitative indicators. Hark and Hofbauer (2018) raised the problem of the quantification of gender equality policies, which also supports their proforma implementation. In Austria, the Federal Ministry of Education, Science and Research currently asks universities to include measures to strengthen gender competence in their performance agreements. This allows universities to include isolated measures like voluntary gender competence or anti-bias training courses for members of appointment committees which are not integrated into a comprehensive strategy. To date, concrete objectives have not been formulated either at institutional or policy level. Possible examples for concrete objectives include the requirement that all members of appointment committees have to participate in an anti-bias training course before the committee starts working or that all lecturers must receive training on gender competent teaching. The implementation of such compulsory training measures could be monitored easily even if this does strengthen the quantification of gender equality policies and does not necessarily depict the change in selection or teaching practices.

The development of monitoring indicators related to the objectives formulated in performance agreements usually takes place in a participatory process. Representatives of the Federal Ministry of Education, Science and Research and the universities discuss concrete proposals for indicators developed by either side. When agreed on, an indicator is included in the regulation for performance agreements and subsequently becomes compulsory for all universities. So all universities have to report the corresponding data on an annual basis. The latest revision of the regulation on performance agreements was carried out in 2019, with supplementary comments published by the Federal Ministry of Education, Science and Research in 2020.

The development of input indicators that focus on the implementation of gender competence measures and indicators to measure gender competence at individual or institutional level are complex endeavors due to the complexity of the underlying construct. They would also represent a further development of the existing set of indicators, which have a lower level of complexity.

An explicit gender competence objective should also be formulated as a requirement in the tasks of university management, and gender competence should be a prerequisite for all rectorate members regardless of their gender. Consequently, it should be a mandatory qualification requirement for rectorate positions and should be verified during the selection process. This would also entail the inclusion of gender competence in training and qualification programs for higher education managers. Making gender competence a general requirement for all rectors and vice rectors would also allow us to challenge the problem raised from a feminist or gender mainstreaming point of view that gender competence is automatically ascribed to women by virtue of their biological sex.

## Footnotes

1 Austria has incorporated gender budgeting into its constitution. Since 2009, the objective of *de facto* equality between women and men in the budgetary planning context is enshrined in the Federal Constitution. Article 13(3), which is applicable to all authorities, states that the “federation, Länder and municipalities have to aim at the equal status of women and men in the budgeting.” Since 2013, gender budgeting must be implemented at the federal level. De facto equality between women and men has to be considered in all stages of administrative action–from the formulation of objectives to their implementation and evaluation.

2 The working group consisted of representatives of higher education institutions, student and staff associations, the Federal Ministry of Education, Science and Research, and gender experts.

3 The Austrian Higher Education Conference (*Hochschulkonferenz*) is a consortium of all higher education institutions in Austria which aims at facilitating cooperation between institutions and formulating common positions for higher education policy.

4 This is an adaptation of a quote from the Canadian Prime Minister Justin Trudeau, whose response to a question regarding the gender balanced and diverse composition of his cabinet was: “Because it is 2015!”.
